# What are the sleep characteristics of elite female athletes? A systematic review with meta-analysis

**DOI:** 10.5114/biolsport.2022.108705

**Published:** 2021-09-30

**Authors:** Kathleen H. Miles, Brad Clark, Peter M. Fowler, Joanna Miller, Kate L. Pumpa

**Affiliations:** 1 Research Institute for Sport and Exercise, University of Canberra, Canberra, Australia; 2 Discipline of Sport and Exercise Science, Faculty of Health, University of Canberra, Canberra, Australia; 3 School of Exercise and Nutrition Sciences, Queensland University of Technology, Brisbane, Australia; 4 AIS Operations, Australian Institute of Sport, Bruce, Canberra, Australia

**Keywords:** Women, Athlete, Sport, Sleep, Women in sport

## Abstract

With the recent growth in female sport, practitioners need to be able to provide specific support to female athletes to ensure their sleep, health and athletic performance are optimised. Examine the patterns, duration and quality of sleep among elite female athletes, and consider the impact of situational challenges and their effects on the sleep of elite female athletes. Data was located through a search of SPORTDiscus, MEDLINE and Scopus from inception up to May 2021. Studies needed to be peer-reviewed research reporting quantitative sleep outcomes for female athletes ≥ 18 years of age and competing at a predefined elite level. A meta-analysis was performed on habitual sleep outcomes (e.g. total sleep time [TST] and sleep efficiency [SE]) measured with actigraphy. A total of 38 studies were included. Meta-analysis showed habitual TST (n = 14) was 7.8 h [7.4, 8.2] (mean [95% CI]), and SE was 86.7% [84.7, 88.6], with high variability among studies (I^2^ = 97.8–98.2%). Subjective sleep complaints are common before a competition, as do post-training sleep disturbances (63% studies report TST decrease), and post-competition sleep disturbances (75% studies report TST decrease). Female athletes achieve satisfactory objective sleep quantity and quality during habitual periods, but experience sleep disturbances pre- and post-situational challenges. There is high variability of objective sleep outcomes, demonstrating the individual nature of habitual female athlete sleep. Overall, future research must focus on optimising the sleep appraisal methods and creating high-quality study designs in a broader number of sports.

## INTRODUCTION

Sleep is an essential requirement for human health, and an integral component in preparation for, and recovery from, athletic training and competition [1]. Important biological processes occur during sleep, including the restoration of immune and endocrine systems, and integral metabolic processes [2]. However, elite athletes may experience suboptimal habitual sleep patterns [3] (i.e. during at home periods under normal living conditions without situational demands) as well as regularly encountering situational challenges such as competition schedules [4] and travel demands [5] that can lead to additional sleep disturbances.

In the general population, polysomnography (PSG) measures suggest females have longer total sleep time (TST), shorter sleep onset latencies (SOL) and better sleep efficiency (SE) than males [6]. Yet females subjectively report poorer sleep than males, including longer SOL and more nocturnal awakenings [7]. Since aspects of objective sleep appear superior in females, other factors may be influencing their subjective sleep ratings, including the misalignment between circadian rhythms (e.g. core body temperature minimum and pineal melatonin secretion) and sleep-wake behaviour [8]; high prevalence of depression and anxiety [9]; and ovarian steroid hormones [10]. Sex differences in sleep are also suggested to increase in magnitude under biological and chronobiological challenges [11], such as the sleep deprivation and travel across time zones routinely experienced by athletes. However, to date, there has been no analysis of how sex differences in sleep interact with the situational challenges imposed on female athletes, and what effect this may have on health, athletic performance and recovery.

Compared to males, females may be more vulnerable to the detrimental effects of sleep loss, showing greater and more prolonged increases of inflammatory biomarkers [12], up-regulation of inflammatory cytokine expression [13] and higher fasting insulin levels [12]. Greater slow wave activity (a measure of sleep propensity) in females has also been observed following sleep deprivation, potentially indicating differences in responses to sleep debt compared to males [11]. Conversely, some evidence indicates females are more resilient to the effect of sleep loss compared with males, expressing lower levels of cortisol following short-term sleep restriction [14], and fewer sleep disturbances after overnight stress [15]. Despite this conflicting evidence, the resulting health impacts of sleep loss in females is more evident with females at an increased risk compared to males for depression and mood disturbance [16] as well as dysfunction of metabolic and cardiovascular processes [17]. Collectively, though current evidence is equivocal, acute or chronically insufficient sleep could have a detrimental effect on the health and athletic performance of female athletes.

With the recent growth in female sport, practitioners need to be able to provide appropriate and specific support to female athletes to ensure their sleep, and thus health and athletic performance are optimised. Despite the sleep differences based on biological sex and the potential impact of sleep loss in females, there has been no systematic appraisal of the current female athlete sleep literature to date. Therefore, the purpose of this systematic review was to: (1) examine the patterns, duration and quality of sleep among elite female athletes; and (2) consider the impact of situational challenges and their effects on the sleep of elite female athletes.

## MATERIALS AND METHODS

The present systematic review with meta-analysis was conducted following the Preferred Reporting Items for Systematic Reviews and Meta-Analyses (PRISMA) guidelines and registered with PROSPERO (CRD42017076869).

### Search strategy and terms

Three electronic databases (SPORTDiscus, MEDLINE and Scopus) were systematically searched from inception up to May 2021 using combinations of the outlined search terms with appropriate truncation and medical subject headings (MeSH). The sleep search terms combined with ‘OR’ were sleep, sleep disturbance, sleep quality, insomnia; the athletic population search terms outlined with ‘OR’ were athlete, sportswoman; and the sex search terms outlined with ‘OR’ were female, woman, women. The search term keywords were combined with ‘AND’ and searched in ‘All Fields’ with the limits of human and English language. To ensure all related texts were captured, the reference lists of included articles were hand-searched.

### Eligibility criteria and selection process

The eligibility of the retrieved records was independently assessed by KHM based on title and abstract. If the information was unclear, the full-text article was screened. Duplicate and irrelevant articles were excluded based on abstract and title by KHM. Articles deemed eligible for full-text review were retrieved and screened against the inclusion criteria by KHM and confirmed by KLP.

Studies were required to meet the following inclusion criteria: (1) the study was published as original research in a peer-reviewed journal as full-text article; (2) data was explicitly reported for female athletes; (3) the female athletes were ≥ 18 years of age; (4) the female athletes were competing at an elite level (defined as Olympic, international, professional, national or Division I collegiate); (5) the study reported quantitative data on sleep outcomes; and (6) the studies did not report data on sleep outcomes following concussion. Only English language studies were included.

### Data extraction and quality appraisal

Data from the eligible studies were extracted by KHM using a predesigned and piloted data extraction form. Only baseline sleep data was used if a study implemented an intervention. ‘Eliteness’ was assessed by applying the taxonomy of Swann and colleagues [18], ranking participants on a continuum (score range 1–16) and allowing categorisations from ‘semi-elite’, through ‘competitive elite’, and ‘successful elite’, to ‘world-class elite’. This taxonomy has previously been employed in systematic reviews of elite athlete sleep [3].

KHM and KLP evaluated the quality of the studies using the Newcastle-Ottawa Scale (NOS) adapted for cross-sectional studies [19]. All score disagreements were discussed and if they could not be agreed upon, were resolved by BC. The adapted NOS has previously been used in systematic reviews of elite athlete sleep [3].

### Statistical analysis

For studies that reported habitual sleep outcomes (i.e. TST, SE, SOL) measured objectively at night (i.e. not naps), random-effects metaanalyses were performed. To normalise effect sizes, the means of eligible studies were transformed with the *measure* argument set to MN (i.e. raw mean) [20]. The I^2^ statistic was used to assess heterogeneity, with I^2^ values of < 25%, 25%–50% and > 50% considered low, moderate and high heterogeneity respectively [21]. Forest plot reference lines were placed at the recommendations of 7 h for TST [22], > 85% for SE [23] and < 20 min for SOL [24]. Metaregression was undertaken to assess if sport, participant number and nights of sleep assessment affected sleep variables (i.e. TST, SE and SOL). The meta-analysis and meta-regression were conducted using the metaphor [20] package 2.4.0 in R Studio version 3.5.0 (“Joy in Playing”).

For studies assessing habitual sleep using subjective methods, and studies exploring the impact of situational challenges to sleep (i.e. competition, training and travel), results are presented qualitatively. This is due to the lack of common sleep assessment methods, reporting standards and outcome variables.

## RESULTS

### Included studies and characteristics

**FIG. 1:**
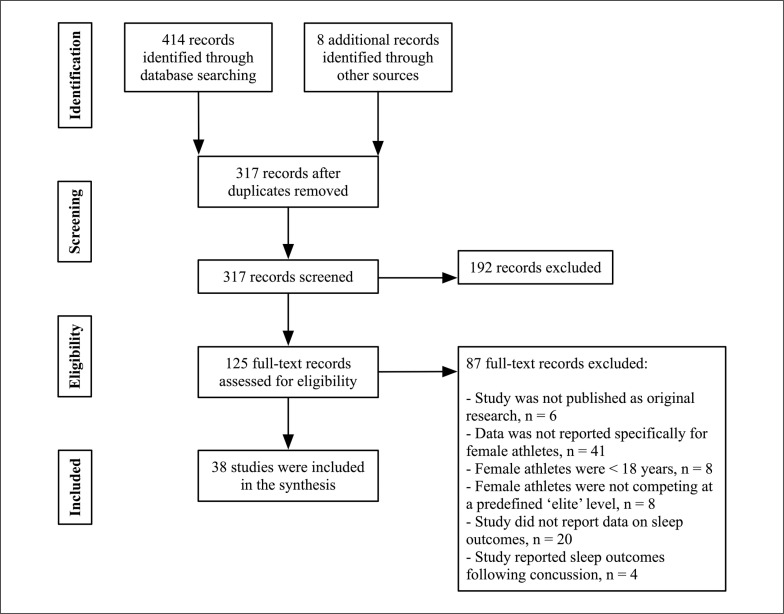
Preferred Reporting Items for Systematic Reviews and Meta-Analysis (PRISMA) flow diagram of each stage of the study selection.

The search strategy returned 422 results (Fig 1). After removal of duplicate (n = 105) and irrelevant articles (n = 192), a total of 125 studies were retained for full-text screening. Following eligibility assessment, 87 studies were excluded as they did not meet the inclusion criteria, leaving 38 studies eligible for review.

Sleep was assessed mainly with subjective methods, with eight studies utilising single questions regarding subjective sleep and nine selection. using the Pittsburgh Sleep Quality Index (PSQI) [25]. Another four studies utilised alternative survey methods to assess sleep. Only one study used an athlete specific questionnaire, the Athlete Sleep Screening Questionnaire (AASQ) [26]. Seven studies assessed sleep solely with objective methods, one using PSG and six using wrist actigraphy. In total, 14 studies used combined objective and subjective assessments of sleep, with 12 using wrist actigraphy and sleep diaries. Two studies implemented PSG in conjunction with subjective sleep quality (one using a visual analogue scale, the other a sleep questionnaire). In regard to actigraphy analysis methodology, ten applied the default device sleep-wake threshold, four specified using the medium threshold, and three did not comment on the threshold used. No study compared subjective and objective sleep assessments.

The evidence quality of the included studies was ‘moderate to high’ (mean score = 6 ± 1 arbitrary unit [AU]), with 82% of studies scoring 5–7 AU (moderate to high quality). Most study designs were observational (n = 33), with five longitudinal and five case-control designs captured. One study controlled for menstrual cycle phase (all participants taking exogenous hormonal contraceptives), two controlled for age, and two controlled for sex. Limited participant descriptions prevented the application of the full Swann taxonomy [18]. Therefore, participants were classified using a modified taxonomy [3] whereby only ‘semi-elite’ or ‘competitive elite’ categories could be judged. Based on this modified taxonomy, 21 studies used ‘competitive elite’ athlete participants (mean total score = 9 ± 4 AU) and 17 studies used ‘semi-elite’ athlete participants. The majority of female athletes were recruited from unspecified multi-sports (n = 10), as well as Australian Rules Football (n = 1), basketball (n = 2), cricket (n = 1), cycling (n = 2), gymnastics (n = 2), judo (n = 1), netball (n = 7), rowing (n = 1), running (n = 1), soccer (n = 7), synchronised swimming (n = 1) and swimming (n = 1).

### Habitual sleep

Sleep characteristics during normal training or out of competition periods were described in 26 studies.

### Quantitative synthesis

**FIG. 2:**
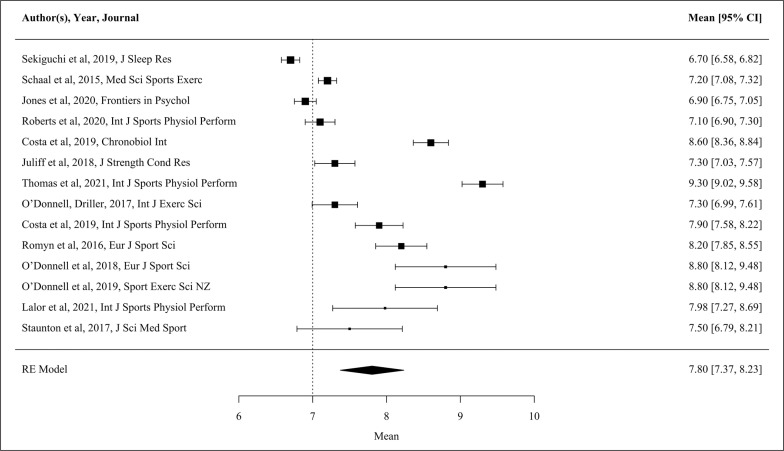
Forest plot showing total sleep time (TST, h) as measured by actigraphy during habitual conditions. The bolder the individual study marker indicates the study was weighted more heavily in the mean outcome. Data presented as mean [95% CI] RE Model, Random Effects Model.

**FIG. 3:**
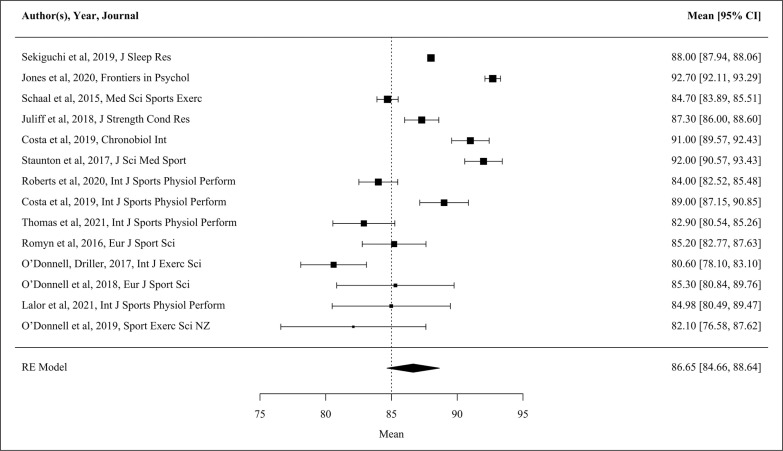
Forest plot showing sleep efficiency (SE, %) as measured by actigraphy during habitual conditions. The bolder the individual study marker indicates the study was weighted more heavily in the mean outcome. Data presented as mean [95% CI]. RE Model, Random Effects Model.

**FIG. 4:**
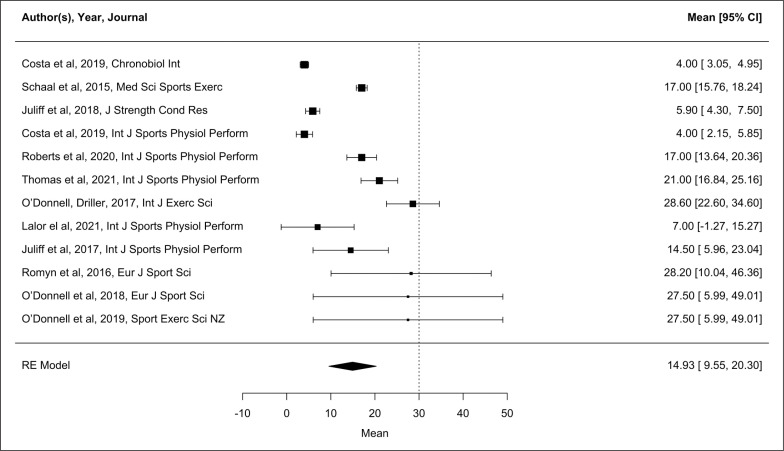
Forest plot showing sleep onset latency (SOL, min) as measured by actigraphy during habitual conditions. The bolder the individual study marker indicates the study was weighted more heavily in the mean outcome. Data presented as mean [95% CI]. RE Model, Random Effects Model.

The majority of habitual sleep studies (n = 16) were conducted using actigraphy. Of these studies, 15 presented data for one or more variables, and therefore, these studies were able to be used in the meta-analysis. Only eight of the included studies recruited ‘competitive elite’ athletes. In addition, based on the NOS assessment [19], two of the studies included in the meta-analysis were high quality. Forest plots of pooled effects are shown in Fig 2–4 (TST, SE and SOL). The mean TST across the included studies (n = 14) was 7.8 h [CI 7.4, 8.2 h]. TST heterogeneity was high (I^2^ = 97.8%). For SE (n = 14) the mean across studies was 86.7% [CI 84.7, 88.6%], again with high heterogeneity (I^2^ = 98.2%). The interaction between the moderators’ sport, participant number and nights of sleep assessment accounts for 48.0% of TST, and 44.6% of SE heterogeneity. The unaccounted sampling variability for the TST and SE actigraphy studies ranges from 4.9–9.9%. From the 12 studies reporting SOL, average onset was 14.9 min [CI 9.6, 20.3 min]. SOL heterogeneity was high (I^2^ = 98.0%). The number of nights of sleep assessment accounts for 26.2% of SOL heterogeneity, with the unaccounted sampling variability 24.6%.

### Qualitative synthesis

Of the habitual studies, one compared the sleep of female athletes to age and a sex-matched control group using self-report, finding triathletes reported sleeping longer than the control group (10 h vs. 7 h) [27]. As part of a study involving multi-sport athletes, Leeder and colleagues compared the sleep of athletes with an age and sexmatched control group [1]. However, the normative sleep data provided did not distinguish female from male athletes when comparing the athletes with the control group. A recent study also compared the sleep of male and female Australian Rules Football (AFL and AFLW) players [28]. Compared to the AFL players, AFLW had less TST (7.9 ± 0.5 vs 7.1 ± 0.6 h, p = 0.000) and lower SE (89.5 ± 2.8 vs 84.0 ± 4.4 %, p = 0.000). Habitual napping practices were examined in five studies [29–33], finding 23.6–35.8% of athletes nap two or more times per week for 0:30–1:00 h [32, 33]. A summary of habitual sleep characteristics assessed using methods other than actigraphy are provided in Table 1.

**TABLE 1:** Habitual sleep characteristics of elite female athletes using methods other than actigraphy

Study	Female athlete n	Nights	Mean values ± SD				
			TST, h	SE, %	SOL, min	Bedtime, hh:mm	Wake time, hh:mm
*Sleep diary*							
Brunkhorst, Kielstein [27]	11	NA	10.3 ± 0.7	NA	NA	NA	NA
Hill et al. [36]	7	7	6.9 ± 0.5	NA	NA	00:00 ± 00:25	NR

*Polysomnography*							
Kinsman et al. [71]	2	4	NR	NR	NR	NR	NR
Silva et al. [41]	60	1	5.6 ± 0.7	89.8 ± 6.5	22.2 ± 23.6	NR	NR
Taylor et al. [57]	7	1	7.5 ± 0.5	NR	19.3 ± 3.5	NR	NR

*Questionnaire*							
Hoshikawa et al. [33]	368	NA	NR	NR	NR	NR	NR
Koikawa et al. [31]	30	NA	7.1 ± 1.2	91.6 ± 8.0	NR	NR	NR
Mah et al. [32]	285	NA	NR	NR	NR	NR	NR
Swinbourne et al. [35]	23	NA	7.1 ± 1.1	NR	NR	NR	NR

*NA* not applicable, *NR* not reported, *SD* standard deviation, *SE* sleep efficiency, *SOL* sleep onset latency*, TST* total sleep time, all results presented as mean ± standard deviation (SD)

Subjective sleep quality and self-reported sleep disturbances during normal training and out of competition periods were described in 17 studies. Five studies [31–35] used the PSQI to assess sleep quality, with four identifying mean global scores above the threshold indicative of ‘poor sleepers’ (> 5 AU [32, 35] and > 6 AU [33, 34]). PSQI sub-category scores also identified 4.3% of athletes experience long sleep latencies and 18.5% have twitching and jerking legs during sleep [33]. One study used the AASQ, finding that soccer players had none to mild sleep problems (4.4–4.7 AU) one month-pre and one-month post competition [29]. Subjective sleep quality was assessed by eight studies using a single question approach. Although the assessment scales and scoring of these questions varied, results indicate athletes experience ‘normal’ to ‘good’ sleep quality over assessment periods ranging from one to twelve days [30, 36–38]. Three studies used other questionnaire methods to assess general sleep disturbance, frequently identifying sleep problems such as difficulty falling asleep, nocturnal waking [39, 40] and waking up tired [41]. A summary table of habitual sleep quality and sleep disturbance is provided in Table 2.

**TABLE 2:** Habitual sleep quality and sleep disturbance of elite female athletes

Study	Female athlete n	Sleep quality	PSQI global score	Prevalence of sleep disturbance
Biggins et al. [29]	20	NA	NA	~ 10–18%
Hill et al. [36]	7	3.5 ± 0.2	NA	NA
Hoshikawa et al. [33]	368	NA	4.7 ± 2.2	32.1%
Jones et al. [34]	12	NA	6.6 ± 1.2	NA
Juliff et al. [46]	157	NA	NA	NR
Juliff et al. [38]	42	2.8 ± 0.5	NA	NA
Juliff et al. [52]	12	NR	NA	NA
Koikawa et al. [31]	30	NA	6.0 ± 4.0	NA
Lalor et al. [59]	11	NR	NA	NA
Lucidi et al. [39]	21	NA	NA	61.9%
Mah et al. [32]	285	7.2 ± 1.5	5.3 ± 2.5	13.0%
Romyn et al. [30]	8	4.0 ± 1.0	NA	NA
Silva et al. [41]	60	NA	NA	50%
Schaal et al. [37]	10	4.5 ± 0.3	NA	NA
Schaal et al. [40]	727	NA	NA	23.9%
Swinbourne et al. [35]	23	NA	8.2 ± 3.3	NA
Taylor et al. [57]	7	NR	NA	NA

*NA* not applicable, *NR* not reported, *PSQI* Pittsburgh Sleep Quality Index, all results presented as mean ± standard deviation (SD) or prevalence (%)

### Effects of competition on sleep

Nine studies used subjective methods to investigate the effect of competition on sleep (Table 3). Two studies (same cohort of participants) reported results of a PSQI assessment pre-competition, with 77.6% of rhythmic gymnasts having a global score > 5 AU (mean score = 7 AU), indicative of poor sleep [41, 42]. The gymnasts who had higher performance ranking scores during competition reported worse sleep quality (mean global score = 8 AU) compared to those with lower scores (mean global score = 6 AU) [41]. One study assessed subjective sleep over three consecutive nights (the night before, the night of, and the night following competition) on 15 separate occasions during a competitive netball season [44]. Subjective TST differed (p < 0.05) between both the night before (8:29 ± 0:44 h) and the night following the game (8:09 ± 0:46 h) compared to game night (6:52 ± 0:40 h) [44]. One study used the athlete specific AASQ to determine sleep problems during competition (implemented on the final day of competition period) in a cohort of University Games athletes [29]. The soccer players in this cohort had a mild AASQ ‘sleep difficulty score’ (5.4 AU [4.2, 6.6]). In addition, approximately 26% of these athletes had moderate to severe sleep problems (i.e. AASQ score between 8–17) during competition. Two studies used the Competitive Sports, Sleep and Dreams Questionnaire, designed to assess sleep habits and disturbances prior (within 12 months) to important competitions [45, 46]. Athletes reported a high prevalence of pre-competition sleep concerns, particularly problems falling asleep (79–83%); waking up early in the morning (29–47%); and nocturnal waking (36–43%) [45, 46]. In contrast, Brandt and colleagues observed 63% of Brazilian athletes experienced good to great sleep quality before important competitions when assessed with a single question metric [47].

**TABLE 3:** Changes to sleep for elite female athletes pre- and post-competition

Study	Female athlete n	Sport, Competition	Sleep disturbance	TST	SE
*Pre-competition*						
Brandt et al. [47]	172	Multi-sport, First day	4.7%	NA	NA
Erlacher et al. [45]	253	Multi-sport, Important	67.9%	NA	NA
Juliff et al. [46]	157	Multi-sport, Olympics	65.9%	NA	NA
Obmiński, Mroczko [53]	5	Judo, Championships	NA	NA	NA
Romyn et al. [30]	8	Netball, Championship	NA	NA	↑*
Staunton et al. [48]	17	Basketball, WNBL	NA	↑**	⟷
Silva, Pavia [42]	67	Gymnastics, World Cup	77.6%	NA	NA
Silva, Pavia [43]	67	Gymnastics, World Cup	78.0%	NA	NA
Swinbourne et al. [35]	23	Rugby 7s and cricket	NA	NA	NA

*Post-competition*						
Biggins et al. [29]	20	Soccer, University Games	~ 26%	NA	NA
Juliff et al. [46]	157	Multi-sport, Olympics	NR	NA	NA
Staunton et al. [48]	17	Basketball, WNBL	NA	↓**	⟷
Obmiński, Mroczko [53]	5	Judo, Championships	NA	NR	NA
Juliff et al. [38]	42	Netball, Championships	NA	↓*	NR
Juliff et al. [52]	12	Netball, International game	NA	↓*	↓**
O’Donnell et al. [44]	11	Netball, In-season	NA	↓*, ↓*	↓, ⟷
O’Donnell et al. [51]	10	Netball, In-season	NA	↓**	↓*
Thomas et al. [50]	10	Soccer, In-season	NA	↓**	↓

*NA* not applicable, *NR* not reported*, PSQI* Pittsburgh Sleep Quality Index, *SE* sleep efficiency, *TST* total sleep time, *WNBL* Women’s National Basketball League, results presented as mean ± standard deviation (SD) or prevalence (%), ↑ increase, ↓ decrease, ⟷ no change

* *p* < 0.05,

** *p* < 0.01

Ten studies using wrist actigraphy provide the most information about the effects of competition on female athletes’ sleep. During an intensive netball tournament, bedtime and wake time were earlier (p = 0.01) and SE greater when compared to normal training (p = 0.03) [30]. In comparison, across two seasons of basketball competition, there was no difference in SE between home compared to away matches, or for regular compared to doubleheader rounds where two games are played in one round of competition [48]. However, on the night before doubleheaders, TST was approximately one hour greater compared with baseline (p = 0.022) and match-day (p = 0.007) [48]. Across a four-week period encompassing five games (three home, two away), 12 basketball players experienced TST of 8.1 ± 1.6 h, SE of 92.0 ± 5.0% and SOL of 30.0 ± 29.0 min [49].

The study by Staunton and colleagues was the first to report on post-competition sleep [48]. Across two seasons, basketball players experienced an 11% reduction in TST the night after doubleheaders compared to regular rounds of competition (p = 0.007) [48]. A recent study from Thomas et al. [50] demonstrated that core (i.e. starting, n = 10) soccer players experienced later bedtimes on the night after a game (+ 37 min; p = 0.032) compared to training nights, alongside reduced sleep duration (−49 min; p = 0.010). Fringe players (n = 8) did not experience any differences between sleep characteristics between the night after a game compared to training nights. In addition to these findings, this study also compared the sleep of the soccer players (n = 18) to an age and sex matched non-athletic control group (n = 18) during a week encompassing the demands of training and competition. In this cohort, the soccer players had greater sleep duration than non-athletes (+38 min; p = 0.009), potentially due to their earlier bedtimes (−31 min; p = 0.047) [50].

Four studies have examined changes to sleep following evening netball competition. During a tournament, the netballers obtained an additional 0:28 ± 0:43 h of sleep (p ≤ 0.05) following afternoon (7:37 ± 1:06 h) compared to evening games (7:08 ± 0:45 h) [38]. Similarly, TST was reduced on the night of a netball game (–1:51 ± 0:58 h, p < 0.05) and the night following a game (–1:05 ± 0:58 h, p < 0.05) compared to the night before a game [44]. Compared to a time and intensity matched training session, the night after a game reduced mean TST (6:03 ± 1:51 h, which was −1:58 ± 1:52 h compared to training; p = 0.008), TIB (8:22 ± 2:16 h, which was −1:34 h compared to training; p < 0.05), and SE (74.4 ± 10.1%, which was −7.7 ± 8.5% compared to training; p = 0.018) [51]. Compared to a time matched rest day, the night after an evening game reduced TST (p = 0.02), lowered SE (p < 0.001), led to early awakenings (waking at 7:08 ± 0:34 h evening game vs. waking at 7:55 ± 0:34 h rest day; p < 0.01) and poorer subjective sleep (p < 0.01) [52].

### Effects of training on sleep

Studies exploring the influence of training on sleep (Table 4) focused on comparing training and rest days (n = 6) or comparing various training loads (n = 3). Judoka had a reduction in subjective sleep quality on training days compared to rest days (p = 0.01) [53]. Comparable results were also reported for netballers, with reduced SE and TST on training days (SE, 82.1 ± 8.9%; TST, 8:01 ± 1:17 h) compared to rest days (SE, 85.3 ± 7.2%; TST, 8:46 ± 1:03 h) [51]. In the three studies specifically comparing evening training and rest days, TST was reduced following evening training compared to a rest day [54–57]. For example, following evening training, soccer players had significantly less TST (7:09 ± 0:28 h, p < 0.001) compared to the night following a rest day (8:35 ± 0:33 h) [54]. As measured by PSG, swimmers experienced 10% more sleep movements at the start of the season (onset; p < 0.05) and 11% more during peak training (p < 0.01) compared to a pre-competition taper [57]. Reported as a percentage of TST, slow wave sleep was greater during onset (28.7 ± 5.1%; p < 0.01) and peak training (29.4 ± 3.2%; p < 0.05) compared to the pre-competition taper (18.4 ± 4.3%) [57]. During intensified training (125% of normal load), synchronised swimmers went to bed 0:49 ± 0:10 h (p < 0.001) later, had reduced TST (−0:21 ± 0:07 h, p = 0.047) and took an additional 0:11 ± 0:05 h to fall asleep (p = 0.053) compared with normal training (no change in self-selected bed and wake times) [37]. There was also a decrease in SE during intensive training (−2.3 ± 1.8%, p = 0.05) [37].

**TABLE 4:** Changes to sleep for elite female athletes under varied training conditions

Study	Female athlete n	Training, nights	Sleep quality	SOL	SE	TST	WASO
*Training vs rest days*								
Costa et al. [54]	17	Evening training, 18	NA	↑ **	⟷	↓ **	↑
Costa et al. [55]	18	Evening training, 8	NA	↑ *	⟷	↓*	⟷
Juliff et al. [46]	157	NA	NR	NA	NA	NA	NA
Obmiński, Mroczko [53]	5	Training days, 15	↓*	NA	NA	NA	NA
O’Donnell et al. [51]	10	Comparable to a netball match, 1	NA	↑	↓	↓	NA
O’Donnell et al. [56]	10	Evening training, 1	NA	↑	↑	↓ **	NA

*Varied training load*								
Juliff et al. [46]	157	NA	NR	NA	NA	NA	NA
Schaal et al. [37]	10	IT, 14	⟷	↑	↓ *	↓ *	↑ *
Taylor et al. [57]	7	Onset, 1; Peak, 1; and Taper, 1	NA	⟷	NA	⟷	↑ *, **

*IT* Intensified training, *NR* not reported, *SD* standard deviation, *SE* sleep efficiency, *SOL* sleep onset latency*, WASO* wake after sleep onset, *TST* total sleep time, ↑ increase, ↓ decrease, ⟷ no change

* *p* < 0.05

** *p* < 0.01

### Effects of travel on sleep

Three studies have investigated the impact of travel on sleep. In a pilot study by Hill and colleagues, subjective sleep was monitored in footballers (n = 7) seven days before, and 12 days following international westward travel [36]. Compared to pre-travel (00:24 ± 00:25 hh:mm), bedtime was earlier on day two posttravel (21:52 ± 00:33 hh:mm, p < 0.05) and later on days three (23:35 ± 00:54 hh:mm) and four (23:08 ± 00:20 hh:mm). Time spent asleep was different from baseline (6:54 ± 0:30 h) compared to day one (7:09 ± 0:22 h) and two post-travel (8:40 ± 0:24 h, p < 0.05), returning to pre-travel durations on days three (6:48 ± 0:44 h) and four (6:21 ± 0:21 h). There was a trend towards poorer quality from day one (3.6 ± 0.5 AU) to four (2.9 ± 0.3 AU) post-travel [36]. Thompson and colleagues demonstrated alterations in subjective sleep quality post-travel [58]. In this study, international football players (n = 20) completed seven hours eastward travel across five time zones. Although no ratings were collected pre-travel, sleep quality worsened following night one posttravel, with the poorest scores reported on the second night [58]. Most recently, Australian cricket players sleep was measured in-flight (business class, travelled west from Melbourne, Australia (GMT + 10) to Chennai, India (GMT + 5.5) crossing 5.5 time zones, with a total travel time of 19 h 35 min), as well as post-flight (i.e. during the tournament upon arrival) [59]. Average in-flight sleep duration was approximately 4.7 h (interpreted from graphical report), with players ranging from ~ 1 h up to 10 h. The sleep obtained during the tournament did not differ between the players who obtained higher versus lower in-flight sleep durations or efficacy.

## DISCUSSION

This review aimed to (1) examine the patterns, duration and quality of sleep among elite female athletes and (2) consider the impact of situational challenges and their effects on the sleep of elite female athletes. Based on a quantitative analysis of the current evidence, female athletes are achieving satisfactory objective sleep quantity and quality during habitual periods, averaging a TST of 7.8 h, SE of 86.7% and SOL of 14.9 min. However, there is particularly high variability of objective sleep outcomes between studies (heterogeneity between 97.8–98.2%), as well as high prevalence of subjective sleep complaints. These findings demonstrate the individual nature of habitual sleep and reinforce the requirement for adaptable and individualised ‘tool-box’ style sleep interventions [60] to be implemented with female athletes. The present review also highlights that female athletes experience post-training, pre-competition and post-competition sleep disturbances and altered sleep-wake patterns.

### Habitual sleep

Studies examined in the present review suggest female athletes attain TST within the recommended duration of sleep for adults (7–9 h) [22], albeit at the middle of these recommendations (Fig 2; actigraphy TST mean [95% CI] 7.8 h [7.37, 8.23]). The present findings are similar to the recent review reporting the pooled TST (7.3 ± 0.7 h) of male and female athletes [3]. However, Gupta and colleagues drew upon a substantially larger total athlete population of predominantly male athletes for habitual characteristics such as TST (n = 1153) [3], making comparisons to the present findings (n = 238) difficult. Meta-analysis indicates that female athletes also experience SE of 86.7% [95% CI 84.7, 88.6], above the threshold which indicates good sleep quality (≥ 85%) [23]. Despite the satisfactory objective sleep quality, female athletes consistently report difficulties falling asleep and nocturnal waking [39, 40]. Comparable subjective sleep problems are associated with psychosocial distress and high inflammatory biomarkers in general population females [12].

The present findings on habitual sleep suggest female athletes obtain satisfactory sleep quantity and quality. However, there are a number of methodological concerns that may influence the interpretation of the current evidence base and must be improved in future studies for practitioners to provide athletes with effective sleep feedback and recommendations. For example, habitual sleep findings are influenced by a lack of consistency and validity in sleep assessment methods. Activity monitors, such as wrist actigraphy, have emerged as a valid alternative to the gold standard of PSG, especially when working with elite athletes in field-based settings [61]. However, they may underestimate sleep duration and overestimate wake duration depending on the sleep-wake threshold applied [61]. Of the studies that used wrist actigraphy, sleep-wake thresholds were briefly described by the majority of studies as being calculated using the default device threshold (typically the medium threshold) [61]. Considering and reporting the choice of sleep-wake threshold in future studies will yield the best combination of the agreement, sensitivity and specificity for the assessment of sleep.

Seven days to three weeks of wrist actigraphy sleep assessment is also necessary to obtain an accurate pattern of sleep that reflects weekly or social rhythms [62]. However, only Staunton and colleagues provided this longitudinal evidence on the habitual sleep of basketball players, indicating they obtained adequate sleep over two seasons [48]. Additional longitudinal observational studies would assist with assessing inter-individual variability in sleep patterns to provide data that is more reflective of ‘normal’ sleep for female athletes. In addition, most of the current habitual sleep evidence is with netball (n = 98 out of 238) and soccer (n = 53 out of 238) athletes. The evidence is, therefore, more of a reflection on the habitual sleep of elite netball and soccer athletes rather than female athletes in general. In male athletes, sleep characteristics appear different between sporting codes (i.e. Australian Rules, rugby union and football), potentially due to varied physiological demands of the sport [63]. Identifying the sleep characteristics of female athletes involved in different individual and team sports will vastly improve upon current evidence and allow tailored support to be provided.

A variety of subjective sleep assessments were used to assess habitual sleep. Subjective measures are inexpensive and simple to implement compared to objective measures and can be useful to monitor changes in the wellbeing of athletes. Preliminary evidence in elite male rugby players indicates subjective measures of sleep duration may be useful, albeit with overestimations in some athletes (by an average of 19.8 min) [64]. However, there is currently no evidence on the effectiveness of subjective sleep assessments in female athletes, nor how subjective assessments compare to objective measures in this population. In addition, the PSQI was used by multiple studies to provide a global measure of sleep quality, as well as subjective TST and SE. However, these results may be misleading, as the PSQI is designed for assessing sleep in the general population, not for subjective sleep screening of elite athlete populations, whose schedules and stressors can vary greatly to the general population [65]. Despite the AASQ being validated for use with athletes for a number of years [26, 65], only one study in the present review implemented an athlete-specific tool [29]. A greater focus on these population-specific tools in future studies would improve the quality of subjective assessments of elite female athlete sleep.

The limited control of menstrual cycle phase also influences the interpretation of habitual sleep findings. Although the impact on sleep architecture itself is currently unclear, evidence suggests hormonal changes throughout the menstrual cycle and exogenous oral contraceptives may influence sleep [10]. In addition, most experimental studies consider menstrual cycle phase due to the potential impact of hormonal fluctuations on human performance (see review [66]). Sleep studies in other populations routinely assess exogenous contraceptive use or determine menstrual cycle phase at the time of sleep assessment [66]. Despite this, only one study within this review has sufficiently controlled for menstrual cycle phase [37]. Menstrual cycle phase and exogenous contraceptive use should be considered in future investigations to improve the interpretation of findings and allow tailored support to be provided.

### Situational challenges

Based on the evidence presented within this review, subjective sleep complaints appear to be common before competition [42, 43, 45, 46]. Conversely, objective sleep assessments indicate improved precompetition SE [30] and TST [38]. Discrepancies between objective and subjective sleep are not unexpected, as these two assessments encompass different aspects of sleep (e.g. SE is a ratio of time spent sleeping to the time spent in bed, while an individual’s subjective sleep quality typically focusses on problems such as initiating or maintaining sleep). There may also be additional factors which contribute to the differences observed in female athletes. Discrepancies in the general population are thought to be related to female’s higher prevalence of depression and anxiety. Similarly, female athletes have been observed to have high levels of anxiety, which is postulated to increase pre-sleep hyperarousal (increased psychological and physiological tension) and lead to perceptions of less restorative sleep [40]. The identified discrepancies between subjective and objective pre-competition sleep in female athletes may pose a problem for practitioners relying solely on subjective sleep monitoring prior to competition. Therefore, it is recommended that practitioners implement complementary objective tools alongside further communication with the athlete to assist with the any sleep concerns.

Sleep disturbances and changes to sleep-wake patterns are evident following double-header rounds of competition [48], evening training [54–56], and following competition [50], especially evening competition [38, 44, 51, 52]. In addition, female athletes may have reduced sleep durations compared to both non-athletic controls [50], and male athletes participating in the same sport [28]. In male athletes, research has demonstrated post-competition sleep loss can impair physiological, cognitive and perceptual recovery [67]. However, direct comparisons between these findings and female athletes is limited due to sex differences in the physiological response to sleep loss [11–13], exercise and recovery (see review [68]). Although this concept was not explored within the studies presented, females tend to have an earlier timing of circadian rhythms (i.e. daily rhythms of body temperature and melatonin secretion are shifted to the left with an earlier peak [8]) and, as such, tend to prefer going to bed and waking earlier than males. High-intensity evening exercise may further advance circadian rhythms of melatonin by approximately 30 min [69]. Therefore, as a result of evening training and competition, female athletes may be sleeping at a later biological clock time [8] compared with male athletes following a comparable stressor. The potential sleep loss and circadian disruption that may occur following evening training and competition, and how this could impair recovery, need to be further investigated in order to be able to optimise female athletes’ athletic performance and health.

### Limitations

The present review did not determine publication bias. Therefore, it is possible that the included studies are a biased sample of all the relevant studies (e.g. the study selection doesn’t include unpublished data and not all data could be presented in summary tables [70, 72, 73]), and the mean effects computed for sleep variables reflect this bias. In addition to this primary limitation, the initial screening of titles and abstracts were conducted by one author, and only articles published in English and in peer-reviewed journals were included. data that is more reflective of “normal” sleep for female athletes. In addition, most of the current habitual sleep evidence is with netball (n = 98 out of 238) and soccer (n = 53 out of 238) athletes. The evidence is, therefore, more of a reflection on the habitual sleep of elite netball and soccer athletes rather than female athletes in general. In male athletes, sleep characteristics appear different between sporting codes (i.e. Australian Rules, rugby union and football), potentially due to varied physiological demands of the sport [63]. Identifying the sleep characteristics of female athletes involved in different individual and team sports will vastly improve upon current evidence and allow tailored support to be provided.

### Key practicl points

- During habitual periods, female athletes are achieving satisfactory objective sleep quantity and quality, yet high prevalence of subjective sleep complaints.
- There is high variability of objective sleep outcomes between included studies, demonstrating the individual nature of female athlete sleep.
- Female athletes are experiencing post-training, pre-competition and post-competition sleep disturbances and altered sleep-wake patterns.
- There are a number of methodological concerns that may influence the interpretation of the current evidence, especially for habitual sleep studies, including: low participant numbers, short duration studies, and limited control of menstrual cycle phase.

## CONCLUSIONS

Based on the current evidence, female athletes are achieving satisfactory objective sleep quantity and quality during habitual periods, however, there is particularly high variability in these sleep outcomes. In comparison, subjective sleep assessments show a high prevalence of habitual sleep complaints. During situational challenges, female athletes are encountering post-training pre-competition and postcompetition sleep disturbances and altered sleep-wake patterns. Despite these findings, there are a number of methodological concerns that may influence the interpretation of the evidence base, especially for habitual sleep studies. This includes a lack of consistency and validity in sleep assessment tools, low participant numbers, short duration studies, and limited control of menstrual cycle phase. The present review also demonstrates the failure of studies to report sleep outcomes stratified by sex and compare sleep outcomes to a general population non-athletic cohort. With the growth in female sport, practitioners must be able to provide appropriate and specific sleep support to female athletes. For practitioners to have the knowledge to provide this tailored support, research must focus on optimising the sleep appraisal methods to create high-quality study designs in a broader number of sports.

## Funding

No sources of funding were received to assist in the preparation of this review.

## Conflicts of interest

Kathleen Miles, Brad Clark, Peter Fowler, Joanna Miller and Kate Pumpa declare they have no conflicts of interest.

## Availability of data and material

Data is available upon request.

## Code availability

Meta-analysis code is available upon request.

## Authors’ contributions

KM contributed to the design of the review, the literature search, data screening and extraction, statistical analyses and managed all aspects of manuscript preparation and submission. BC, PF and JM all contributed to the design of the review and to writing and editing of the manuscript. KP contributed to the design of the review, data screening and extraction, and contributed to writing and editing of the manuscript. All authors meet the criteria for authorship.
